# Low-Cost Pseudo-Anthropomorphic PVA-C and Cellulose Lung Phantom for Ultrasound-Guided Interventions

**DOI:** 10.3390/gels9020074

**Published:** 2023-01-17

**Authors:** Natalia Arteaga-Marrero, Enrique Villa, Ana Belén Llanos González, Marta Elena Gómez Gil, Orlando Acosta Fernández, Juan Ruiz-Alzola, Javier González-Fernández

**Affiliations:** 1 Grupo Tecnología Médica IACTEC, Instituto de Astrofísica de Canarias (IAC), 38205 San Cristóbal de La Laguna, Spain; 2 Departamento de Neumología, Complejo Universitario de Canarias (HUC), 38320 San Cristóbal de La Laguna, Spain; 3 Departameto de Radiología, Complejo Universitario de Canarias (HUC), 38320 San Cristóbal de La Laguna, Spain; 4 Instituto Universitario de Investigaciones Biomédicas y Sanitarias (IUIBS), Universidad de Las Palmas de Gran Canaria, 35016 Las Palmas de Gran Canaria, Spain; 5 Departamento de Señales y Comunicaciones, Universidad de Las Palmas de Gran Canaria, 35016 Las Palmas de Gran Canaria, Spain; 6 Departamento de Ingeniería Biomédica, Instituto Tecnológico de Canarias (ITC), 38009 Santa Cruz de Tenerife, Spain

**Keywords:** polyvinyl alcohol cryogel, cellulose, phantom, ultrasound, biopsy, training

## Abstract

A low-cost custom-made pseudo-anthropomorphic lung phantom, offering a model for ultrasound-guided interventions, is presented. The phantom is a rectangular solidstructure fabricated with polyvinyl alcohol cryogel (PVA-C) and cellulose to mimic the healthy parenchyma. The pathologies of interest were embedded as inclusions containing gaseous, liquid, or solid materials. The ribs were 3D-printed using polyethylene terephthalate, and the pleura was made of a bidimensional reticle based on PVA-C. The healthy and pathological tissues were mimicked to display acoustic and echoic properties similar to that of soft tissues. Theflexible fabrication process facilitated the modification of the physical and acoustic properties of the phantom. The phantom’s manufacture offers flexibility regarding the number, shape, location, and composition of the inclusions and the insertion of ribs and pleura. In-plane and out-of-plane needle insertions, fine needle aspiration, and core needle biopsy were performed under ultrasound image guidance. The mimicked tissues displayed a resistance and recoil effect typically encountered in a real scenario for a pneumothorax, abscesses, and neoplasms. The presented phantom accurately replicated thoracic tissues (lung, ribs, and pleura) and associated pathologies providing a useful tool for training ultrasound-guided procedures.

## 1. Introduction

Ultrasound (US) imaging has been increasingly employed for lung evaluation despite the impedance mismatch between the pleural lining and the air inside the lung [1,2]. Although the detection of the wave propagation is limited to the superficial tissue, US imaging provides valuable clinical insight into patients affected by pleural diseases, subpleural consolidations, and interstitial lung diseases (ILD) [1]. Lung ultrasonography has been employed to evaluate various disorders such as pneumothorax, peripheral lesions, consolidations, and diaphragm-related disorders [2]. Currently, the COVID-19 pandemic resulted in the adoption of a US-based approach to limit thecontamination and exposure of clinical practitioners [3]. Thus, US imaging was established as a suitable technique for diagnosis and monitoring of acute respiratory findings.

For ILD, the lung parenchyma becomes fibrotic and stiff, leading to dyspnea, dry cough, and respiratory failure. However, diagnosis at an early stage is complicated since the symptoms are not specific [2]. US is increasingly used to guide vascular access procedures, but its safety, effectiveness and usefulness considerably depend on the operators’ skills [4]. Consequently, clinical practitioners in thoracic US imaging require the means to obtain diagnostic and therapeutic capabilities in the assessment of diverse pathologies, in which infectious and neoplastic etiology diseases present a high prevalence. Additional training is requiredto develop proficiency in cognitive and psychomotor skill sets [5] in order to reduce the failure rate and to improve patient safety [6,7].

The use of phantoms for training US-guided interventions provides an effective educational tool [8]. The ideal phantom should be inexpensive, simple to construct, versatile, easily repairable, reproducible, and reusable. The phantom should have a long shelf life, present no infection issues, and be composed of nonperishable materials. Furthermore, the phantom should reproduce the texture and resistance of human tissues, as well as approximate the same ranges in the speed of sound, attenuation, and scattering coefficients [9,10]. A close match with the speed of sound is particularly important as this determines the distances in the US image [9]. Phantoms mimicking lung tissue are commercially available from several manufacturers, including (CIRS, Inc. [11], CAE Healthcare [12], and Kyoto Kagaku Co., Ltd. [13]). These are usually employed for training and the investigation of the effects of the breathing motion in the planned dose distributions, four-dimensional imaging, deformable image registration, and radiotherapy delivery techniques [14,15,16,17]. Other purposes include the verification of Monte Carlo simulations and the validation of quantitative measurement software [18,19,20]. Currently, more realistic and improvedphantoms are required for emerging imaging systems that provide multimodal and multiparametric data acquisition [21]. However, the limitations of replicating the complex geometry and structure result in phantoms that represent a simplified anatomy with a narrow range of accessible tissue structures [22,23]. Most importantly, the high purchase costs prevent their routine use as training tools. Thus, inexpensive and yet flexible custom-made phantoms are a reasonable solution for research institutions to overcome this issue [4].

Traditionally, dampened natural sponges were commonly employed to emulate the inner structure of the lungs [24,25]. In this manner, A- and B-lines can be nicely reproduced in US images [1,14]. Although this solves the cost problem, the instability over time presents a serious drawback, since these structures either present a short lifespan or are not deformable. Therefore, a more advanced approach was recently introduced using 3D printing technology, which has become more accessible, versatile, and accurate [21]. However, 3D-printed materials may be avoided in US-guided imaging as bulk material for the lung, since the speed of sound or attenuation are not accurately replicated [26,27]. In any case, 3D-printed materials can be employed for specific phantom’s parts such as the ribs.

For US imaging, commercial phantoms specifically developed for vascular access are available. However, these are difficult to alter, expensive, and degrade with use [28]. In addition, a limited lifetime is expected due to the tracks produced by repeated needle insertions. Consequently, custom-made phantoms have become an affordable alternative [4] with an associated order of magnitude cost reduction. Such phantoms, intended for training personnel in US diagnostic and interventional procedures, have previously been reported in the case of the liver [23], heart [26], and kidney [29]. In some cases, although a realistic phantom was successfully fabricated, the speed of sound was inaccurate [23]. Acoustic specifications from the Quantitative Imaging Biomarker Alliance (QIBA), established by the Radiological Society of North America (RSNA), require a speed of sound of 1540 ± 20 m/s and an attenuation coefficient of 0.6 ± 0.2 dB/cm·MHz for standardized tissue-mimicking phantoms [30].

Thus, a phantom based on polyvinyl alcohol cryogel (PVA-C) and cellulose for biomedical applications is described herein. Polyvinyl alcohol (PVA) is a hydrophilic and biocompatible polymer. This material forms a hydrogel, known as PVA-C, when crosslinked after a freeze–thaw cycling process. PVA-C shows textural, mechanical, and elastic properties that can be tuned to closely match those of soft tissues [31,32,33]. In addition, it is easily customized at a low cost. Hence, PVA-C is an attractive candidate for manufacturing phantoms for biomedical and medical device applications [34]. These PVA-C phantoms provide properties quite similar to that of biological tissues and present a high structural rigidity, longevity, and resistance to crack formation compared to other commonly employed materials, such as agar or gelatin [35]. The additional advantages of PVA-C-based phantoms are their biocompatibility and hydrophilicity. Nevertheless, the fabrication process requires a precisely controlled temperature and a long post-manufacturing time depending on the number of freeze–thaw cycles required [35].

PVA-C phantoms were previously employed for US imaging to investigate the elastic properties of healthy and pathological soft tissues [31,36]. However, PVA-C is a weak acoustic attenuator [32,37]. So the addition of scattering particles is required to produce a strong attenuation and provide a speckle pattern. Additionally, these particles control the acoustic properties, such as the speed of sound, the attenuation, and the backscatter coefficients. N-propanol and propylene glycol modify the speed of sound; whereas evaporated milk, cellulose, or graphite vary the attenuation [38]. A uniform mixture of the bulk material and the scattered particles is required to produce a rapidly varying dynamic speckle field [39]. Cellulose is a renewable, biocompatible, nontoxic, and biodegradable natural biopolymer. It is easily mixed and provides replicable results, in comparison to glass beads, when employed as scatterers in PVA-C phantoms [31,35,39]. Hydrogels based on cellulose are attractive biomaterials for multidisciplinary fields due to their excellent properties [40]. In addition, some cellulose derivatives have been used or even considered suitable for biomedical applications [41]. Recently, low-cost custom-made bimodal phantoms based on PVA-C and cellulose were introduced for microwave and US medical applications [35]. These phantoms demonstrably fulfilled the acoustic specifications for soft tissues using a flexible fabrication process with off-the-shelf materials. Thus, cellulose is presented as a suitable scatterer material for PVA-C phantoms.

In the present work, an easily reproducible, durable, and deformable lung tissue phantom is presented. This pseudo-anthropomorphic phantom mimicked the healthy parenchyma and some of the lungs’ commonly found pathologies as embedded inclusions made of gaseous, liquid, or solid materials. Moreover, fine needle aspiration biopsy (FNA) and core needle biopsy (CNB) were successfully performed. Thus, the capabilities of the phantom were demonstrated as a training tool for the acquisition and improvement of the technical skills associated with thoracic diagnostic and US-guided interventions.

## 2. Materials and Methods

Phantoms mimicking the healthy parenchyma were fabricated using PVA-C (99% hydrolyzed, molecular weight 89,000–98,000, Sigma Aldrich, St. Louis, MO, USA) and cellulose (Cellulose microcrystalline, 20 μm, Sigma Aldrich) as US scattering material, at 10% and 1% concentrations, respectively. Benzoic acid (Sigma Aldrich) was added as a preservative at a 0.1% concentration. The corresponding percentages required for the mixture were calculated in weight/weight (*w*/*w*), and the materials were employed as received. The dimensions of the phantoms were 165 mm × 75 mm × 50 mm (width × depth × height) corresponding to the container used for fabrication and storage. The strength and elasticity of the phantoms were controlled by the concentration of the PVA-C as well as the number of freeze–thaw cycles required to crosslink the polymer. An increment in PVA-C concentration provides a harder constraint than an increment in the number of freeze–thaw cycles. For the healthy parenchyma, a single freeze–thaw cycle was employed since an adequate and realistic elasticity was provided.In addition, a suitable support was provided for the different inclusions. Further details regarding the fabrication process can be found in [35,42].

The healthy parenchyma phantoms were upgraded by randomly embedding both spherical and irregular shape inclusions at variable depths. These inclusions were filled with gaseous, liquid, or solid materials to mimic the diverse pathologies of interest. For the gaseous and liquid inclusions, a customized shell was fabricated with a bubble-shape polydimethylsiloxane (PDMS) (Polytek Development Corp) film employing the lost-wax technique [43]. PDMS is a popular material with excellent optical transparency that has been previously used in microfluidics and artificial organ systems [29]. Cellulose at a 1% concentration was added since this material is anechoic to the US scanner. Nonporous PDMS walls were required to prevent the gas or liquid leakage, which could compromise the shelf life of the phantom over the long term. The gaseous inclusions were filled with air during the fabrication process, whereas the liquid ones were filled with saline solution. Nevertheless, it is possible to fill these inclusions with any other liquid such as distilled water or retinol. These two types of inclusions were mainly employed to mimic hypoechoic lesions that may produce shadowing of the underlying structures. The solid inclusions were fabricated using the same PVA-C concentration selected for the healthy parenchyma. However, an additional freeze–thaw cycle was required to insert these inclusions into the phantom. A higher cellulose concentration (2%) was also used. These inclusions mimicking hyperechoic lesions were clearly distinguishable from the healthy parenchyma in the US images. The elasticity was maintained despite the extra freeze–thaw cycle.

**Figure 1:**
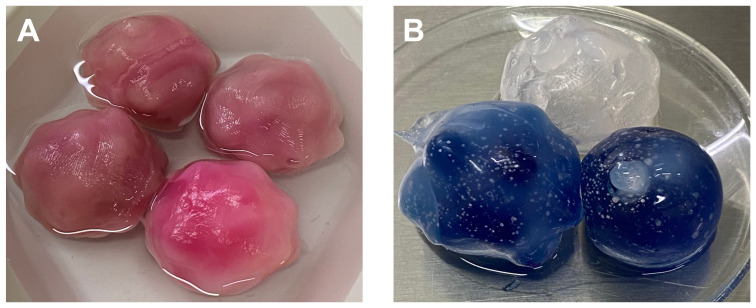
The fabricated inclusions before being embedded into the PVA-C block mimicking the healthy parenchyma. (**A**) PVA-C solid inclusions with irregular shape and color coded. (**B**) Gaseous (transparent) and liquid (blue) inclusions with irregular and spherical shapes fabricated with PDMS.

Furthermore, a color code was established to assess whether the FNA and CNB were successfully performed. Standard food coloring, red and blue, was used to fill the solid and liquid inclusions, respectively. The gaseous inclusions were transparent as was the PDMS shell. Figure 1 displays the fabricated inclusions.

**Figure 2:**
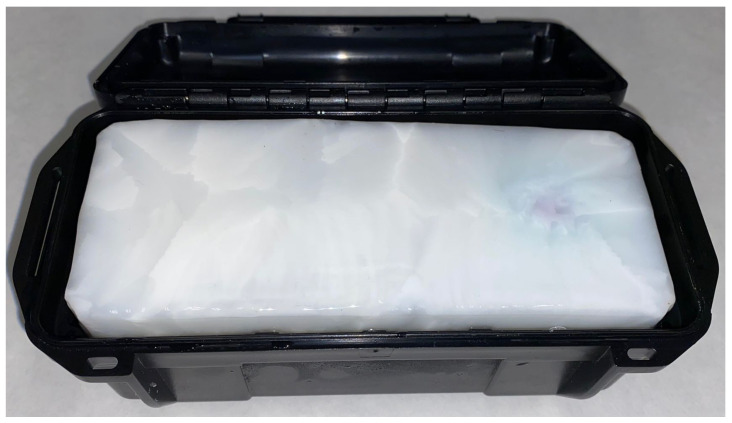
The fabricated phantom with embedded inclusions placed in the storage container. Dimensions: 165 mm × 75 mm × 50 mm (width × depth × height).

In addition, ribs were 3D-printed using polyethylene terephthalate and embedded within the phantom. The phantom offered flexibility regarding the number, shape, location, and composition of the inclusions, as well as the presence of the ribs and pleura. This structure was mimicked by a bidimensional reticle made of PVA-C. This PVA-C was mixed with glass beads (iM16K, 3MTM) until saturation was reached. Figure 2 shows the fabricated phantom in the storage container. As can be observed, the phantom mimicking the healthy parenchyma was white, the color of the crosslinked PVA-C. Overall, the estimated time for each phantom fabrication was approximately between 48 and 72 h. The variationsin fabrication time were related to the final size of the phantom because a slow and controlled thawing was preferred. The number and typeof embedded inclusions also affected the fabrication time. Regarding the fabrication of these inclusions, once the mold was available, the solid inclusions were the longest to process (around 24 h). As mentioned above, this was due to the extra freeze–thaw cycle required to crosslink the PVA-C.

## 3. Results and Discussion

### 3.1. Ultrasound Characterization

The speed of sound and the acoustic attenuation are critical phantom properties [44]. In soft tissues, the average speed of sound, commonly assumed by clinical US scanners, is 1540 m/s [33], specifically, 1540 ± 20 m/s as required by QIBA [30]. The speed of sound should match this reference value, as closely as possible, since deviations induce image blurring due to non-adjusted beam forming [45]. The acoustic properties of the phantoms are usually measured by a well-established method, through-transmission ultrasonic spectroscopy [45,46,47,48]. The measured speed of sound for the fabricated phantoms was within the limits of soft tissues reported in the literature, as well as within the standards required by QIBA [10,30,33]. As previously demonstrated, a single freeze–thaw cycle at 10% PVA-C concentration provided a good match for soft tissues. The average speed of sound reported was 1540.3 ± 0.3 m/s and 1540.2 ± 0.1 m/s for the 1% and 2% cellulose concentrations, respectively [35].

The attenuation coefficient for soft tissues ranges between 0.5 and 3.3 dB/cm·MHz [10], with 0.6 ± 0.2 dB/cm·MHz the value required by QIBA [30]. A 10% PVA-C and 1% cellulose concentration provided a realistic speckle noise in the US images, although the measured attenuation coefficient was 0.030 ± 0.003 dB/cm·MHz [35]. Higher attenuation was achieved by increasing the cellulose concentration, since its modification has reportedly no significant effect in the speed of sound [31]. Thus, 2% cellulose concentration approximately doubled the attenuation coefficient to 0.071 dB/cm·MHz [35]. This value was within the range previously reported for PVA-C [49]. An extra increment in cellulose concentration would match the required attenuation values. As mentioned above, this is a feasible option provided by the flexibility of the fabrication process. Different materials, such as sucrose, introduce inhomogeneities that increase the realism of the phantom and further modify its acoustic properties [35,42].

### 3.2. Ultrasound Imaging

US characterization of the fabricated phantom was based on the images acquired using a standard portable US system (MicrUs EXT-1H, Telemed UAB). The acquired images confirmed the similarity of the fabricated phantoms to healthy lung parenchyma, as well as the embedded inclusions to common pathologies. This qualitative assessment was performed by a panel of experts composed of radiologists and pulmonologists. In order to compare these images to a real case scenario, the largest publicly available lung US dataset was employed [50,51]. This dataset is composed of images and videos of several lung pathologies as well as healthy controls.

**Figure 3:**
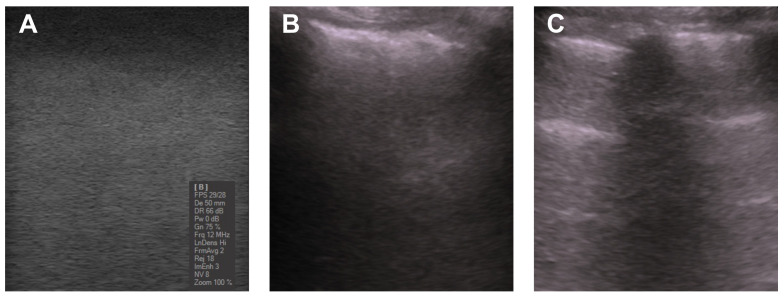
(**A**) US image of the fabricated PVA-C phantoms mimicking the healthy parenchyma. (**B**) Lung US of a subject with pleural thickening on the underlying healthy parenchyma as well as the shadow caused by the ribs. (**C**) Lung ultrasound of a healthy subject showing the A-lines typically observed in the normal parenchyma. The details of the acquisition for the images (**B**,**C**) can be found in [50,51].

**Figure 4:**
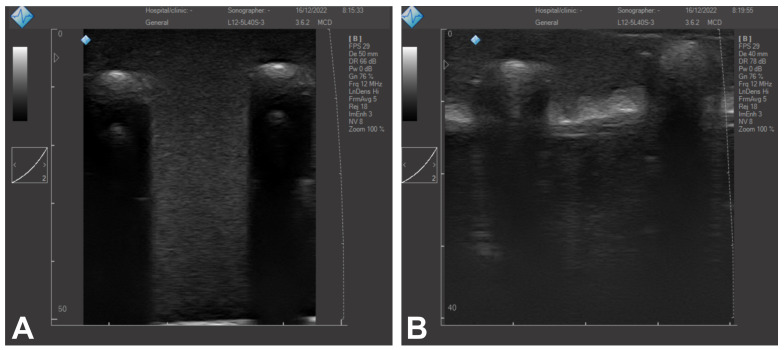
US image of the fabricated PVA-C phantoms including the ribs without (**A**) and with (**B**) mimicked pleura.

For illustrative purposes, images showing the mimicked healthy lung parenchyma are displayed in Figure 3A. The lung US images of a subject are shown in Figure 3B,C. The lung surface is typically smooth for a healthy subject, whereas an unsmooth and heterogeneous surface indicates a pathology [2]. As can be seen in the images, the healthy lung parenchyma has a homogeneous speckled appearance similar to that of real tissues. Figure 4 shows a more detailed US image of the phantoms containing the 3D-printed ribs. In addition, the images illustrate the difference when the pleura is included (Figure 4B).

**Figure 5:**
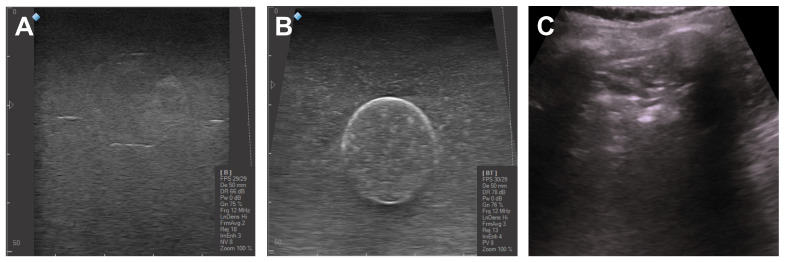
US images of solid inclusions embedded in the PVA-C phantom (**A**,**B**) as well as a real lung ultrasound (**C**). These PVA-C solid inclusions mimic the healthy parenchyma and demonstrate the flexibility of the fabrication: (**A**) with an irregular shape (10% PVA-C and 1% cellulose) and (**B**) with a spherical shape (12.5% PVA-C and 2% cellulose). (**C**) Lung ultrasound of a subject exhibiting a consolidation caused by pneumonia. The details of the acquisition for image (**C**) can be found in [50,51].

**Figure 6:**
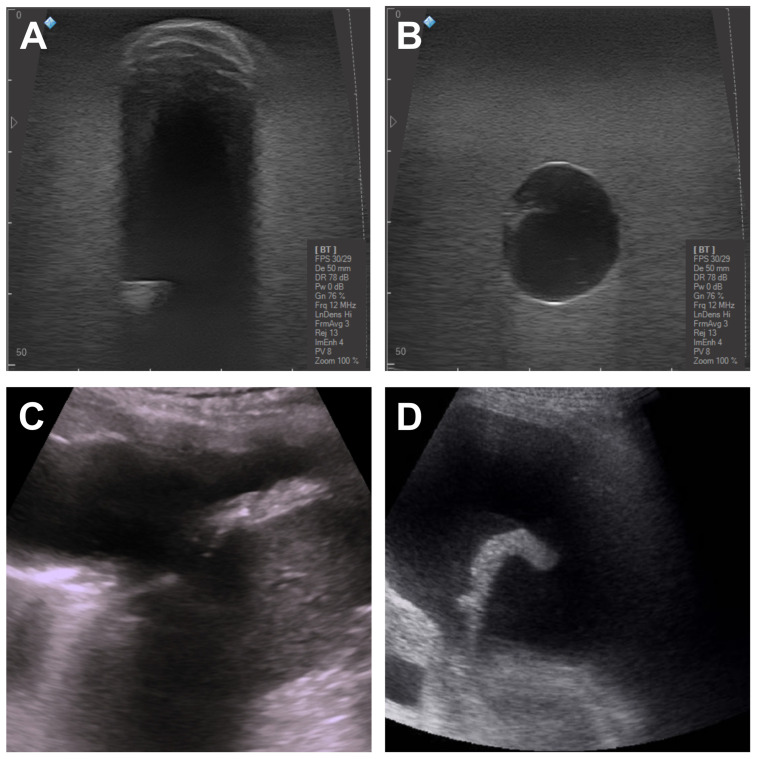
US images of gaseous and liquid inclusions embedded in the PVA-C phantom (**A**,**B**) as well as real lung ultrasounds (**C**,**D**). (**A**) An air-filled inclusion and (**B**) a liquid inclusion filled with 0.9% saline solution. (**C**) Lung ultrasound of a subject affected by pleural effusion. (**D**) Pleural effusion with compressive atelectasis of the lung caused by effusion. The details of the acquisition for the images (**C**,**D**) can be found in [50,51].

Several embedded inclusions are displayed in Figure 5 and Figure 6. In all the images, the healthy parenchyma is seen as a homogeneous background and the respective inclusions as hyperechoic or hypoechoic regions. Two solid inclusions are shown in Figure 5A,B, respectively, to demonstrate the flexibility of the fabrication process. Their main difference is the concentration of cellulose, 1% versus 2%, as well as the border shapes, either irregular or spherical. The boundaries of the inclusion in Figure 5A are barely noticed as compared to the parenchyma since its composition is similar. The additional freeze–thaw cycle required seemed to solely change the consistency of the inclusion. Furthermore, in Figure 5B, the accumulation of scatter from the parenchyma can be noticed on the top of the inclusion. The real scenario is displayed in Figure 5C. These solid inclusions resemble neoplasia, necrotizing pneumonia, abscesses, as well as lung consolidations [52,53,54]. Gaseous and liquid inclusions embedded on the phantoms are shown in Figure 6A,B. Figure 6C,D display real scenarios; that is, the lung US of a subject affected by pleural effusion and a liquid inclusion with internal tracks, respectively. The gaseous inclusion mimics a pneumothorax (Figure 6A). The liquid inclusion (Figure 6B) presents an echogenicity similar to that observed when pleural effusion occurs, although a laminar pattern is normally observed [54,55]. The small white region inside this inclusion resembles fibrinous layers that appear as partitions within the pleural effusion [56], as depicted in Figure 6D. Moreover, the inclusions made of PDMS, specifically the liquid ones, exhibited a strong signal at the outer surface as can be noticed in Figure 6B.

As mentioned above, although in a real scenario, the geometry of the displayed inclusions may not exactly correspond with that exhibited by the mentioned pathologies, these inclusions resemble their appearance and characteristic artifacts in the US images. At the phantom’s surface, the material was rigid enough to resist the deformation caused by the US transducer as in clinical practice. Additionally, no special care was required to maintain the integrity of the phantom’s surface.

An e-video (Video S1) is included as additional material. Different types of embedded inclusions as well as their location are shown by sweeping through the surface of one of the fabricated phantoms.

### 3.3. Invasive Surgical Procedures

The capabilities of the presented phantom were tested by experienced pulmonologists. In-plane and out-of-plane needle insertions were performed under US image guidance with different gauge needles. The content of the gaseous inclusions was extracted and refilled several times. The resistance of these inclusions to the needle insertion, and the recoil effect noticed, were similar to that experienced in a real scenario for a pneumothorax as determined by the experts. In this case, the needle produced barely visible tracks. This inclusion was refilled after several punctures. However, after leaving the phantom in storage for a few minutes, the gaseous inclusion was compressed and emptied.

A 21G needle was employed to perform FNA on the liquid inclusions. Similar to that observed before, these inclusions could be emptied and refilled several times. However, as opposed to the gaseous ones, most of the liquid remained within the inclusion after a few days in storage. For this needle gauge, the residual needle tracks were minimally visible.

**Figure 7:**
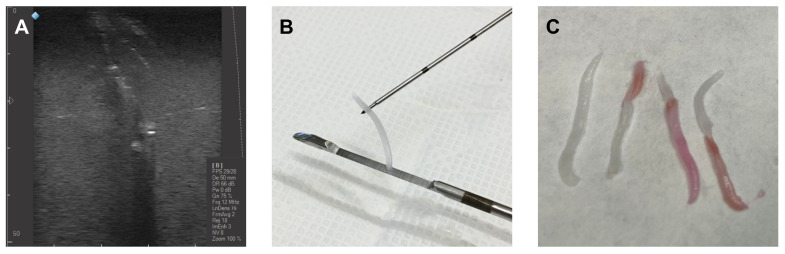
Coarse needle biopsy (CNB): (**A**) 18G needle tracks produced on the phantom after performing several CNBs on a solid inclusion. (**B**) Material extracted from the phantom when the CNB fails. The extraction of white material indicates that the biopsy was performed at an unsuitable depth, before reaching the inclusion or completely puncturing it. (**C**) Material extracted in subsequent CNBs. The level of success can be quantified by the proportion of light red material extracted, since this indicates the distance travelled within the phantom and the inclusion, before performing the biopsy.

An 18G needle was employed to perform CNB on the solid inclusions. For this gauge, the needle tracks were clearly visible as shown in Figure 7A. The material extracted from the CNB procedure is also shown (Figure 7B,C). After a few days in storage, the tracks were refilled with liquid by diffusion minimizing the impact of these tracks on the US images. Therefore, less visible tracks were observed. Several CNBs were carried out on the same inclusion to demonstrate plausible scenarios regarding the level of success of the procedure, as illustrated in Figure 7C. The color code used gives an account of the distance travelled within the phantom before performing the biopsy. Ideally, healthy and pathological tissues are necessary for further comparative analysis. Furthermore, the resistance to the needle insertion of the solid inclusion was similar to that typically encountered in vascular access procedures for the healthy parenchyma, abscesses, and neoplasms.

An e-video (Video S2) is included as additional material showing the CNB procedureon a solid inclusion.

### 3.4. Comparison to Commercial Phantoms

Commercially available US phantoms present a simplified anatomy, coarse morphology, and relatively high price for training a limited number of people [23]. In addition, some models only demonstrate a single pathology, and they are not dynamic [57]. The costs usually depend on the country and provider, but standard prices are in the order of a few thousand EUR [23]. Furthermore, the costs increase up to several tens of thousand EUR when provided with anthropomorphic detailed alternatives. The biopsy insertion requires sticking the corresponding needle into the phantom. This inherently destructive use may permanently damage the phantom, since many repeated insertions are performed for training purposes. The estimated number of biopsies achievable before ending the lifetime of the phantom depends on the end-user. Several models are currently available and have been reported in the literature for US-guided interventional procedures. The Blue Phantom series (CAE Healthcare, Sarasota, FL, USA) [58] offers multiple training opportunities. These include, among others, the lumbar puncture and spinal epidural training model, the abscess drainage training model, as well as the thoracic US model available for teaching US-guided pleural effusion assessment and drainage. In addition, the Zerdine©-based Image-guided Abdominal Biopsy Phantom (CIRS, Norfolk, VA, USA) [59] provides the means to visualize biopsy insertions producing minimal needle tracking during the lifetime of the phantom. The manufacturer specifies that debris and air bubbles entrained in the gel may cause some permanent tracking. Thus, higher gauge, wetted, and de-aired needles are recommended. Alternatively, the Kyoto lumbar trainer and the ABDFAN© (Kyoto Kagaku Co., Ltd., Kyoto, Japan) can also be found [60,61].

### 3.5. Comparison to Custom-Made Low-Cost Phantoms

Custom-made phantoms are the simplest solution to the cost problem of the commercial phantoms for US-guided vascular access. In this case, the fabrication is based on common and cheap materials. Three basic components are required: one to provide bulk, another to simulate US scatter, and a third to represent targets [62]. Regarding the bulk material, custom-made phantoms have been reported based on gelatin, agar, ballistic gel, tofu, and meat, including pork and chicken breast as cadaveric models of vascular access [20,28,57,63,64,65,66]. Particularly for the lung, sponges are also a common alternative [1]. The 3D-printed and low-cost alternative, based on more durable materials, is an exception since an adequate phantom could cost one or two orders of magnitude below those commercially available worldwide. Nevertheless, the use of 3D-printed materials prevents the performance of the required biopsy for training. Meat-based phantoms provide a more realistic scenario and have a closer echogenicity to that of human tissue. Non meat-based phantoms often have low background echogenicity that enhances needle visibility [28,66]. For this reason, scattering agents are usually employed for non meat-based phantoms. These included sugar-free Metamucil, flour, cornstarch, calcium carbide, silicium carbide, sugar-free psyllium, graphite particles, glass microspheres, reticulate foam, and cellulose [35,62,63,67]. Regarding the targets, a basic requirement is a clear distinction from the surrounding medium in the US images. However, the difference in acoustic impedances should not produce reverberations [9]. In addition, custom-made phantoms should ideally provide different levels of complexity and, if possible, be easily modulated [66]. Typically reported materials for targets have included silicon tubes, balloons, pipette bulbs, vegetables, pasta, etc., [62].

Of course, all these approaches present serious drawbacks, but there must be a tradeoff between the associated costs and the application at hand. For instance, needle track artifacts after repeated punctures are less visible in meat-based than in gelatin-based phantoms. Food-based phantoms require special storage conditions, and instability over time is a serious drawback [66]. In addition, meat-based phantoms, in certain situations, must be avoided due to the risk of the transmission of pathogens from uncooked meat. Further details regarding the advantages and limitations of available simulators for training US-guided procedures can be found in [9,28].

In summary, the proposed phantom exhibited several advantages that included longer durability, robustness, and reproducibility when compared to the above mentioned alternatives as well as to animal organ models for US-guided vascular access [29,65]. In addition, the phantom provided a great flexibility in terms of the internal structures that could be mimicked. Most importantly, the performance of biopsies was feasible. The phantom described in the present work can be reproduced in most labs. In order to do so, the only requirement is that of a standard chemical lab, its basic infrastructure, and the off-the-shelf materials. The price range of the phantom is in the same order of the 3D-printed and low-cost alternatives, approximately a few hundred EUR. As most available low-cost alternatives, the presented phantom mimicked healthy and pathological lung tissues without the pulmonary airways. Future work will be dedicated to obtain deformable lung phantoms and tackle one of the major challenges in thoracic radiotherapy [14]. In addition, the development of a fully anthropomorphic lung phantom will be considered. Thus, more specific and geometrically adjusted internal structures will be included.

## 4. Conclusions

A low-cost pseudo-anthropomorphic phantom was presented in which healthy lung parenchyma as well as pathologies of interest were mimicked. This phantom was easily replicable due to the simplicity and versatility of the fabrication process withoff-the-shelf materials namely PVA-C, cellulose, and PDMS. The inexpensive, deformable, and long shelflife phantom pres mechanical characteristics and acoustic properties similar to the soft tissues targeted. The acoustic properties were easily tuned by varying the concentration of its constituents and the number of freeze–thaw cycles. The previously reported average speed of sound and attenuation coefficient for 10% PVA-C and 1% cellulose were 1540.3 ± 0.3 m/s and 0.030 ± 0.003 dB/cm·MHz, respectively. This provided a realistic speckle noise in the US images. A 2% cellulose-concentration phantom reached values of 1540.2 ± 0.1 m/s and 0.071 dB/cm·MHz for the speed of sound and attenuation, respectively. The qualitative assessment of the US images confirmed the similarity of the fabricated phantoms, with and without inclusions, to healthy as well as pathological tissues. Additionally, the performance of multiple biopsies was feasible, as well as the possibility of emptying and refilling the gaseous and liquid inclusions several times. FNA and CNB procedures were performed under US guidance demonstrating the capabilities of the phantom for training purposes. The availability of this low-cost phantom offers an interesting tool to improve the ability to use thoracic diagnostic US imaging and US-guided interventions. Improvements are expected in the diagnosis and precision in therapeutic interventions for pathologies such as thoracentesis, endothoracic drainage placement, FNA, and CNB.

## Data Availability

Not applicable.

## Supplementary Materials

The following supporting information can be downloaded at: https://www.mdpi.com/article/10.3390/gels9020074/s1, Video S1: US Sweeping through the Fabricated Phantom. An e-video shows the fabricated phantom. Different types of embedded inclusions can be observed by US sweeping through the surface. Notice that the fabricated ribs were extracted for the recording to facilitate the visualization of all the embedded inclusions; Video S2: CNB on a Solid Inclusion. An e-video shows a CNB procedure on a solid inclusion. The e-video was recorded with the ribs extracted to facilitate the visualization.
